# A report of the Social Vital Signs Workshop at WONCA Asia Pacific Regional Conference 2019

**DOI:** 10.1002/jgf2.304

**Published:** 2020-02-18

**Authors:** Toshihiro Terui, Junki Mizumoto, Yukinori Harada, Akira Ohya, Yuko Takeda

**Affiliations:** ^1^ Center for Medical Education and Career Development Fukushima Medical University Fukushima Japan; ^2^ Department of Family Practice Ehime Seikyou Hospital Ehime Japan; ^3^ Department of Diagnostic and Generalist Medicine Dokkyo Medical University Hospital Tochigi Japan; ^4^ Division of General Medicine Mimihara general hospital Osaka Japan; ^5^ Division of Medical Education Faculty of Medicine Juntendo University Tokyo Japan

## Abstract

We, Team SAIL, held the workshop at WONCA APR Conference 2019 for presenting the concept of Social Vital Signs (SVS) to an international audience.

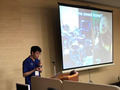

To the editor

Medical professionals now recognize that understanding and addressing social determinants of health (SDH) is vital for clinicians to help individual patients improve their health and well‐being.^1^, ^2^ However, in the setting of busy daily practice, obtaining such information is often disregarded.^3^ Therefore, we have developed a screening tool that provides a practical and easy‐to‐use framework in clinical practice for identifying the social needs of patients. We call it “social vital signs (SVS).” We have held workshops to introduce SVS as a way of identifying patients in social need to healthcare professionals at various conferences and meetings held by Japan Primary Care Association. The SVS framework has been broadly accepted as a useful tool to address SDH by participants and has been implemented in their practices.^4^

In June 2019, we held the workshop at WONCA Asia Pacific Regional Conference entitled “The ‘social vital signs’ mnemonic to improve awareness about determinants of health” (Figure 1). This was the first time we presented the concept of SVS to an international audience. A total of 24 participants attended this workshop. Most of them were family physicians or general practitioners. They were from Greenland, Singapore, Hong Kong, Macau, and Japan. We began the session with a short presentation to introduce SDH and the concept of SVS. We then received feedback about SVS from the participants’ standpoint with consideration to the structural differences in each country, such as geography, demographics, and economic circumstances. One junior doctor from Greenland stated that social issues to be considered by care providers were enormous because of the harsh climate and depressed economy. He also mentioned the difficulties of securing access to healthcare services in an extremely large country with a low‐density population. Several others mentioned the usefulness of SVS in teaching medical students and residents. Since medical schools in many countries have curricula about learning SDH, the potentiality of SVS as an educational tool should be explored.

**Figure 1:**
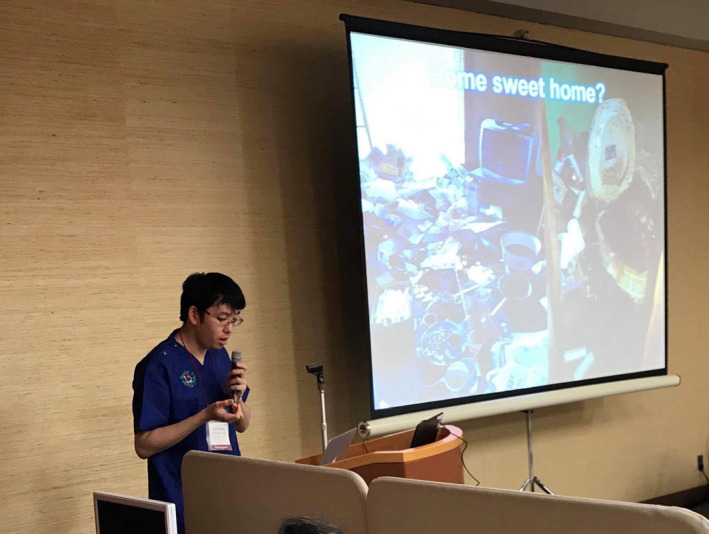
The view of our workshop in WONCA APR Conference 2019

There was also a discussion about using SVS as a scoring tool to measure the level of complexity of patients’ social situations. We agreed that SVS was not fit for numerical assessment nor does it provide criteria to differentiate the patients’ needs. Instead, it is a tool to probe the SDH of individual patients. By the end of our workshop, many participants expressed their willingness to utilize and promote SVS as an effective tool for primary care providers. At the same time, some pointed out the importance of taking an upstream approach^1^, ^2^ by recognizing the “causes of causes” of poor health along with individually meeting the patients’ nonmedical needs.

In summary, SVS was presented at an international conference for the first time and was well perceived by participants as a quick and easy tool to identify patients’ social needs in various practice settings. SVS was also recognized as being useful for teaching medical students learning SDH. Further research is required to assess the practical value of SVS with possible setting‐based modifications and to explore wider implementation and dissemination to healthcare providers.

## CONFLICT OF INTERESTS

The authors have stated explicitly that there are no conflicts of interest in connection with this article.
